# Association of a Total Cholesterol Polygenic Score with Cholesterol Levels and Pathological Biomarkers across the Alzheimer’s Disease Spectrum

**DOI:** 10.3390/genes12111805

**Published:** 2021-11-17

**Authors:** Nathalie I. V. Nilsson, Cynthia Picard, Anne Labonté, Theresa Köbe, Pierre-François Meyer, Sylvia Villeneuve, Daniel Auld, Judes Poirier, for the PREVENT-AD Research Group, Alzheimer’s Disease Neuroimaging Initiative

**Affiliations:** 1 Department of Psychiatry, McGill University, Montreal, QC H3A 0G4, Canada; nathalie.nilsson@mail.mcgill.ca (N.I.V.N.); theresa.koebe@gmail.com (T.K.); pierre-francois.meyer@mail.mcgill.ca (P.-F.M.); sylvia.villeneuve@mcgill.ca (S.V.); 2 Douglas Research Centre, Montreal, QC H4H 1R3, Canada; cynthia.picard@mail.mcgill.ca (C.P.); anne.labonte@affiliate.mcgill.ca (A.L.); 3 Centre for the Studies in the Prevention of Alzheimer’s Disease, Montreal, QC H4H 1R3, Canada; 4 McGill Centre for Integrative Neuroscience, Montreal Neurological Institute, McGill University, Montreal, QC H3A 0G4, Canada; 5 McConnell Brain Imaging Center, Montreal Neurological Institute, McGill University, Montreal, QC H3A 0G4, Canada; 6 Genome Centre, McGill University, Montreal, QC H3A 0G4, Canada; daniel.auld@mcgill.ca; 7 Department of Medicine, McGill University, Montreal, QC H3A 0G4, Canada

**Keywords:** polygenic score, Alzheimer’s disease, cholesterol, amyloid, tau protein, aging

## Abstract

Midlife hypercholesterolemia is a well-known risk factor for sporadic Alzheimer’s disease (AD), and like AD, it is highly influenced by genetics with heritability estimates of 32–63%. We thus hypothesized that genetics underlying peripheral blood total cholesterol (TC) levels could influence the risk of developing AD. We created a weighted polygenic score (TC-PGS) using summary data from a meta-analysis of TC genome-wide association studies for evaluation in three independent AD-related cohorts spanning pre-clinical, clinical, and pathophysiologically proved AD. APOE-ε4 variant was purposely included in the analysis as it represents an already well-established genetic risk factor for both AD and circulating TC. We could vastly improve the performance of the score when considering *p*-value thresholds for inclusion in the score, sex, and statin use. This optimized score (*p*-value threshold of 1 × 10^−6^ for inclusion in the score) explained 18.2% of the variance in TC levels in statin free females compared to 6.9% in the entire sample and improved prediction of hypercholesterolemia (receiver operator characteristics analysis revealed area under the curve increase from 70.8% to 80.5%). The TC-PGS was further evaluated for association with AD risk and pathology. We found no association between the TC-PGS and either of the AD hallmark pathologies, assessed by cerebrospinal fluid levels of Aβ-42, p-Tau, and t-Tau, and 18F-NAV4694 and 18F-AV-1451 positron emission tomography. Similarly, we found no association with the risk of developing amyloid pathology or becoming cognitively impaired in individuals with amyloid pathology.

## 1. Introduction

Alzheimer’s disease (AD), dementia, and cognitive impairment are multifactorial in nature, whose cause and progression are typically influenced by a combination of risk factors such as old age, genetics, and lifestyle factors [1]. Approximately half of the AD phenotypic variance is explained by genetics; however, most of the genetic variants are unidentified [2], as is the mechanism by which they act.

One of the suggested lifestyle factors involved in AD is the level of blood total cholesterol (TC) [1], as well as LDL content. For example, several studies have shown that high TC levels, specifically in midlife, are associated with an increased risk of developing AD [3,4,5,6,7,8], yet others have shown little or no association [9,10,11]. Moreover, higher circulating cholesterol levels (TC or LDL cholesterol) have been associated with increased amyloid load [12,13,14] and hypometabolism in brain regions affected by AD [3]. Late-life TC levels have also been examined, but again with contradictory results. In a study with nursing home residents, levels of blood TC were found to be significantly increased in pathologically defined AD patients, compared to individuals free from AD pathology [15,16]. Similarly, when compared to non-demented subjects with atherosclerotic heart disease, TC levels were found to be increased in individuals with possible clinical or probable AD [17]. Contrary to these findings, one study found that TC levels were decreased in AD individuals compared to controls [18].

A factor that could partly explain these variable results is the fact that AD is a clinicopathological construct [19], and a clinical diagnosis of probable AD has a sensitivity of 81% and a specificity of 70% to predict definite AD (pathophysiologically proven) [20]. This has very recently led to a proposal of new guidelines for the definition of AD in research settings by the NIA-AA Research Framework [21]. These guidelines propose that, for research purposes, AD should be defined as a biological construct determined by the presence of pathology as assessed with the *A/T/N* classification system, depending on levels of amyloid-β (Aβ, A), phosphorylated TAU (p-Tau, T) and neurodegeneration (N) [22]. When pathology data is not available, proxies of the aforementioned pathologies have been developed using cerebrospinal fluid (CSF) or positron emission tomography (PET) measurements. Of note, this biological definition was proposed to also work with current clinical diagnoses of AD; e.g., AD neuropathological change with or without accompanying cognitive decline. In this study, we have used the *A/T/N* framework to define participants according to their neuropathological status and to refine their clinical statuses (healthy or AD).

Similar to AD, TC levels are also markedly influenced by genetics [23,24,25]. For example, heritability is estimated to be 58–79% for AD [26] and 32–63% for TC [27]. Considering the genetic background of both conditions and the fact that they are linked in terms of risk, it is possible that some of the genetic variance seen in AD can be explained by variants influencing blood cholesterol levels. The best example is the APOE-E4 allele, which serves both as a very significant risk factor of sporadic AD as well as a potent modulator of TC in the blood.

Along these lines, an early study did investigate the effect of a TC polygenic score (TC-PGS) in AD but failed to reveal any significant effects [28]. However, only patients with clinically defined AD were investigated in this paper. In addition, scores were based only on genome-wide significant single nucleotide polymorphisms (SNPs) compared to performing an evaluation of the best *p*-value cut-off, and the polygenic score only explained a small portion of the variance (3.6%) in cholesterol levels. It is thus possible that the inclusion of low effect loci in the score and using the new classification system for AD could reveal important associations.

The aims of this study were to first examine multiple TC-PGSs to determine the effect of inclusion of low effect loci and, at the same time, evaluate the influence of factors such as sex and statin use. Secondly, after determining the score with the best prediction, we aimed at investigating the TC-PGS in the context of AD as a biological construct, examining associations with the *A/T/N* pathologies and cognition in individuals with AD pathology.

We show, using three different AD-related cohorts that cover the pre-symptomatic to the symptomatic end-stage of the disease, that despite creating an improved TC-PGS, no associations with either pathology or cognition could be detected.

## 2. Materials and Methods

### 2.1. The Meta-Analysis Summary Data

Summary statistic data from the Global Lipids Genetics Consortium’s meta-analysis (GLGC) of TC GWAS’s [24] was downloaded from csg.sph.umich.edu/willer/public/lipids2013/ (downloaded on 21 June 2018). Results from the joint analysis of metabochip and GWAS data were used. Before being used for scoring, ambiguous SNPs were excluded, and only SNPs present in all three target data sets were kept. The details of this data set are described elsewhere [24]. Briefly, this data are based on 63 blood total cholesterol genome-wide association studies (GWAS’s) for a total of 114,230 individuals [24]. 48 vs. 15 studies were of European and non-European ancestry, respectively. The ratio of women in these studies ranged from 0 to 76.8%, and the mean age in the studies ranged from 16 to 75 years. Most studies investigated individuals free of lipid-lowering drugs (44/63) and the majority of studies had a fasting regime before cholesterol measurements (51/63). Raw data contained 2,446,981 SNPs, whereof 15.4% were ambiguous. These were excluded resulting in a data set with 2,069,037 SNPs.

### 2.2. Study Populations

#### 2.2.1. PREVENT-AD

The Pre-symptomatic Evaluation of Novel or Experimental Treatments for Alzheimer’s Disease (PREVENT-AD, openpreventad.loris.ca/, accessed on 26 January 2018) cohort, based at the Centre for Studies on the Prevention of AD in Montreal, Canada (StoP-AD, douglas.research.mcgill.ca/stop-ad-centre, accessed on 26 January 2018), is a longitudinal study of older, healthy individuals (55+) with a parental or multiple-sibling history of AD [29]. Data for all variables were obtained from data release 5.0 (30 November 2017) except for APOE genotype, PET, and genetic data. For these variables, the latest available data at the center was used to be included in future data releases. Each participant and study partner provided written informed consent. All procedures were approved by the McGill University Faculty of Medicine Institutional Review Board and complied with the ethical principles of the Declaration of Helsinki. In this cohort, 382 individuals were genotyped and selected for evaluation. Of these, 41 were excluded during quality control procedures and 35 were excluded due to lack of data for covariates and target phenotypes, resulting in a final data set of 306 individuals.

#### 2.2.2. ADNI

Data used in the preparation of this article were obtained from the ADNI database (adni.loni.usc.edu, accessed on 3 December 2015). The ADNI was launched in 2003 as a public-private partnership led by Principal Investigator Michael W. Weiner, MD in the US. The primary goal of ADNI has been to test whether serial magnetic resonance imaging, PET, other biological markers, and clinical and neuropsychological assessment can be combined to measure the progression of MCI and early AD. For up-to-date information, see www.adni-info.org (accessed on 3 December 2015). For this study, a subset of ADNI consisting of individuals with genetic data and a family history of AD (first-degree relative affected) was used. All data, but the CSF data, which were downloaded on 22 June 2018, were downloaded on 3 December 2015. The final data set, after quality control and merging of data (see below; Genetic data—Merging of ADNI data), contained 1065 individuals. These were further filtered for having a family history of AD, resulting in a data set of 401 individuals.

#### 2.2.3. ROSMAP

ROSMAP consists of two longitudinal clinical–pathologic cohort studies of aging and AD from the Rush Alzheimer’s Disease Center in the US (www.radc.rush.edu/, accessed on 28 May 2019) [30]. In this study, a subset containing individuals with genetic and pathology data was used. Briefly, 1081 individuals passed genomic quality control steps and were used for scoring. Of these, 547 had phenotype data and were thus selected for the final data set.

### 2.3. Genetic Data

#### 2.3.1. Quality Control

QC procedures [31] for the genetic data was done similarly for all cohorts and were performed in PLINK v1.9 [32,33] as follows: heterozygous haploid genotypes excluded, sex check, relatives excluded (identity by descent > 0.1875), sample and genotyping call rate > 0.95, minor allele frequency > 0.05, and Hardy–Weinberg equilibrium < 1 × 10^−6^. SNPs were then matched with the GRCh37 genome (www.ncbi.nlm.nih.gov/assembly/GCF_000001405.13/#/st, accessed on 28 May 2019). A principal component analysis with 1000 Genomes phase 3 data as a reference [34,35] was performed to determine ancestry and filter for individuals with European ancestry. Briefly, long-range linkage disequilibrium regions and ambiguous SNPs were first excluded from 1000 Genomes data and then merged with target cohort genetic data. Any ambiguous SNPs from target data were then excluded. Merged data were pruned with a sliding window of 2000 bp with a step size of 200 bp, excluding SNPs with an R2 > 0.2 (--indep-pairwise 2000 200 0.2), and PCs calculated (--pca) in PLINK [32,33]. Averages and standard deviations of PC1 and PC2 for Europeans in 1000 Genomes were determined, and target cohort individuals were determined to be of European ancestry if their PC1 and 2 fell within ± 3 SD of the 1000 Genomes cohort means. PCs were then calculated again within the European target cohorts to include as covariates in subsequent analyses.

#### 2.3.2. Imputation

PREVENT-AD, a subset of ADNI (“ADNI 1 GWAS” data set, see below), and ROSMAP data were imputed using the Sanger Imputation Service [36] (imputation.sanger.ac.uk/, accessed on 3 December 2015). Briefly, quality-controlled genetic data was uploaded and pre-phased with SHAPEIT2 [37] and imputed with positional Burrows–Wheeler transform [38] using the 1000 Genomes cohort [34,35] as a reference panel. Only post-imputed SNPs with an info score greater than 0.7 were kept (similar to [39]) to balance the quantity of excluded data (14% in [39]) with data quality.

#### 2.3.3. Merging ADNI Data

Two genomic data sets were used for ADNI; the “ADNI 1 GWAS” data set genotyped using the Illumina Human610-Quad BeadChip, and the “ADNI WGS” data set genotyped using a whole-genome sequencing platform. Before merging, both data sets were quality controlled and “ADNI 1 GWAS” data was imputed. Some individuals were present in both data sets, in which case data from the “ADNI WGS” data set was used. The merged genetic data set contained 6,164,853 SNPs and 1065 individuals.

### 2.4. TC-PGS

Polygenic scoring was done with PLINK [32,33]. Using the PREVENT-AD cohort, SNPs were clumped with a sliding window of 250 kbp and filtering all SNPs with a linkage disequilibrium R2-value > 0.1 (--clump). Multiple weighted PGS’s (using summary statistics β-values) were then calculated (--score) at various *p*-value cut-offs (1 × 10^−100^, 1 × 10^−50^, 1 × 10^−40^, 1 × 10^−30^, 1 × 10^−20^, 1 × 10^−10^, 1 × 10^−8^, 1 × 10^−7^, 1 × 10^−6^, 1 × 10^−5^, 1 × 10^−4^, 1 × 10^−3^, 1 × 10^−2^, 0.05, 0.1, 0.5, and 1).

### 2.5. TC and Hypercholesterolemia Measurements

In PREVENT-AD, TC levels were assessed in plasma drawn from non-fasting individuals at the eligibility visit (i.e., before baseline measurements). In ADNI, TC levels were assessed in whole blood drawn at the screening visit from fasting individuals. ADNI TC measurements were transformed from mg/dL to mM to match the PREVENT-AD data by dividing values with 38.67. ROSMAP was not used for blood TC analyses. A hypercholesterolemia variable was created for PREVENT-AD and ADNI, by assuming that all individuals on statins and all non-treated individuals with TC levels > 6.2 mM were hypercholesterolemic [4].

### 2.6. CSF Measurements

In both PREVENT-AD and ADNI, CSF was obtained by lumbar puncture following an overnight fast. Levels of Aβ-42, p-Tau, and t-Tau were measured by the Innotest^®^ ELISA (enzyme-linked immunosorbent assays, Fujirebio) [40] and the Roche Elecsys CSF immunoassays (data file UPENNBIOMK9_04_19_17.csv) [41,42], for PREVENT-AD and ADNI, respectively. Of note, the Elecsys Aβ-42 CSF immunoassay is currently under development for investigational use only and has an upper technical limit of 1700 pg/mL. Values above this limit are based on extrapolation of the calibration curve, and the performance of these values has not been formally established. These are still included in this study. In PREVENT-AD, Aβ-40 levels were further assessed by the MSD^®^ MULTI-SPOT Assay System (V-PLEX Plus Aβ Peptide Panel 1 (6E10) Kit, MesoScale, Rockville, USA).

### 2.7. PET Imaging

PET scans were performed in PREVENT-AD using fluorine 18-labeled NAV4694 and AV-1451 (Flortaucipir, Montréal, Canada) to estimate the deposition of Aβ and TAU in the brain, respectively. Standardized uptake value ratios (SUVR) were computed by dividing tracer uptake by cerebellar gray matter uptake (Aβ) or by inferior cerebellar gray matter uptake (TAU). For details on PET procedures, see [13].

### 2.8. Amyloid Positivity Status

According to the recently proposed biological definition of AD, we categorized individuals as on or off the AD spectrum by the presence of amyloid pathology in the brain [21,22]. In PREVENT-AD, individuals were split into amyloid negative (Aβ(−)) and positive (Aβ(+)) status based on Aβ PET values (Aβ(+) defined as SUVR > 1.37), similar to McSweeney and colleagues [43]. In ADNI, we used the CSF p-Tau/Aβ-42 ratio as a proxy for brain amyloid pathology, as described by Hansson et al., [44]. Briefly, the CSF values were extracted from the last available visit for each individual, and a ratio ≥ 0.028 was considered as Aβ(+) and thus on the AD spectrum, whereas a lower ratio was considered Aβ(−). In ROSMAP, semiquantitative estimates of post-mortem neuritic plaque density as recommended by the Consortium to Establish a Registry for Alzheimer’s Disease (CERAD score) were used to define Aβ(+) individuals. This is a four-point scale, and individuals, where scores three or four were considered Aβ(+).

### 2.9. Statistical Analyses

All statistical analyses were performed in R [45]. Data was handled with the “data.frame” [46] and “tidyverse” [47] packages and plotted with the “cowplot” [48] package. For a full list of data sets, software, and r packages used, and their respective links, see Supplementary Table S1. Values are reported as mean ± standard error or the mean (SE) if not otherwise stated.

#### 2.9.1. Descriptive

Differences in cohort characteristics, such as age, sex, and TC levels, were analyzed with either a Welch two-sample t-test (comparing two cohorts) or an ANOVA (comparing the three cohorts) for continuous variables and with Pearson’s chi-square test for categorical variables. Post-hoc analysis was performed if primary analyses were significant, and comparisons were between all three cohorts. Here, Tukey HSD was used for continuous variables and post-hoc chi-square test was used for categorical variables. R package “psych” [49] was used to compute summary statistics.

#### 2.9.2. TC Levels and *p*-Value Thresholding

The relationship between each score and blood TC levels was evaluated with a linear regression with genetic PCs 1–10, age, age^2^, APOE-ε4 status, sex, and statin use as covariates. Additional R2 explained were calculated as the difference in R2 between a model containing only the covariates and a model containing covariates and the TC-PGS. Standard deviations at each cut-off were determined by bootstrapping (*n* iterations = 5000) using the R package “boot” [50,51] and R2-values were calculated using the “rcompanion” package [52]. Effects of statin use and sex on the relationship between the TC-PGS and TC levels were assessed by stratification, first by statin use and then by sex (in statin-free individuals). The TC-PGS that explained most of the variance were selected for further analyses in all three cohorts with interactions terms for sex and statin use when sample sizes allowed.

#### 2.9.3. Hypercholesterolemia ROC Analyses

Discrimination of hypercholesterolemic from healthy individuals was evaluated by ROC curve analysis and quantified by the AUC using the “pROC” package [53]. Data were stratified for sex, and the difference between a model containing the covariates (PCs 1–10, age, and age^2^) and a model containing covariates plus the TC-PGS was evaluated with DeLong’s test.

#### 2.9.4. CSF and PET Linear Regression Analyses

Each dependent variable was examined for distribution patterns and transformed if not normally distributed and analyzed with multiple linear regression. In PREVENT-AD, models were corrected for genetic PCs 1–10, age, APOE-ε4 status, statin use, and run with a sex*TC-PGS interaction term. In ADNI, the same covariates were used except for statin that was included in the interaction term (statin*sex*TC-PGS). Aβ PET data were not normally distributed, even after transformation, and individuals were therefore analyzed both with a robust regression (same model as above) and by linear regressions after stratifying for Aβ(+) status. The Aβ(−) group had a sufficient sample size to be analyzed with the aforementioned model (*n* = 80), whereas the sample size of the Aβ(+) group was too small to run the same regression (*n* = 18). Thus, the regression was run with age, APOE-ε4 status, statin use, and sex as covariates and only investigated the main effect of the TC-PGS.

#### 2.9.5. Risk of AD and Cognitive Impairment Logistic Regression Analyses

We evaluated whether the TC-PGS associated with the risk of ending up on the AD spectrum is defined as being Aβ(+) by logistic regression. Cognition was analyzed in ADNI and ROSMAP, and these analyses were limited to Aβ(+) individuals to investigate individuals on the AD spectrum only. In both ROSMAP and ADNI, CI was defined as having a clinical diagnosis of either MCI, AD, or other dementia. Risks between the TC-PGS and both Aβ(+) status and CI were evaluated by multiple logistic regressions. All models were corrected for genetic PCs 1–10, age, and APOE-ε4 status. In PREVENT-AD, statin use was further included as a covariate, and the model was run with a sex*TC-PGS interaction while statin use was included in the interaction term in ADNI. Similar models were used in ROSMAP but without the statin factor as this data were not available.

#### 2.9.6. Conversion Rate

The effect of TC-PGS on conversion rate in ADNI and age of onset in ROSMAP was evaluated with Kaplan–Meier survival analysis [54]. Before filtering, the TC-PGS was categorized into tertiles (i.e., low, medium, and high TC-PGS). In ADNI, individuals that were Aβ(+) with either no CI or with an MCI diagnosis at baseline were selected. Follow-up time ranged from three to 120 months. Conversion was defined as developing a clinical diagnosis of AD. In ROSMAP, Aβ(+) individuals were selected, and the conversion was defined as receiving a clinical diagnosis of either possible or probable AD. A larger sample size in ROSMAP allowed for stratification on sex. Analyses were done using the “survival” package [55,56], the “ggfortify” package [57,58] were used for plotting and the “survminer” package [59] was used for creating survival tables.

## 3. Results

### 3.1. Cohort Characteristics

Three AD-related target cohorts were evaluated in this study: the Pre-symptomatic Evaluation of Experimental or Novel Treatments for Alzheimer’s Disease (PREVENT-AD, *n* = 306) [29], Alzheimer’s Disease Neuroimaging Initiative (ADNI, *n* = 401), and Religious Orders Study and Rush Memory and Aging Project (ROSMAP, *n* = 547) [30]. They differed in their percentages of females (70, 48, and 71%, respectively; *p* = 4.9 × 10^−14^), apolipoprotein E ε4 allele carriers (*APOE*-ε4, 37, 57, and 24%, respectively; *p* = 1.5 × 10^−23^) and statin-treated individuals (23 and 51% for PREVENT-AD and ADNI, respectively; *p* = 3.8 × 10^−13^, Table 1). Post-hoc analyses revealed that the proportion of females was higher in PREVENT-AD and ROSMAP compared to ADNI (p’s ≤ 1.0 × 10^−8^) and that the proportion of *APOE*-ε4 carriers was significantly different between all cohorts (p’s ≤ 5.0 × 10^−7^). Age was recorded at the different assessments (blood, CSF, PET, amyloid positivity (Aβ(+)) status and cognition), was different between the cohorts in all instances, and were significantly higher in PREVENT-AD (5.42 ± 0.06 mM and 5.09 ± 0.06 mM, respectively, *p* = 9.3 × 10^−5^).

#### Global Lipids Genetics Consortium

Summary data were matched with the target cohorts. After matching, the proportion of non-ambiguous SNPs present in each cohort was 86.4, 89.7 and 91.3% for PREVENT-AD, ADNI and ROSMAP, respectively. After filtering SNPs not present in all the data sets, 1,653,356 SNPs remained, representing 67.6% of the original number of summary data SNPs (see Supplementary Figure S1 for Manhattan plots of included and excluded SNPs).

### 3.2. Amount of Variance Explained in TC Blood Levels by TC-PGS

**Figure 1:**
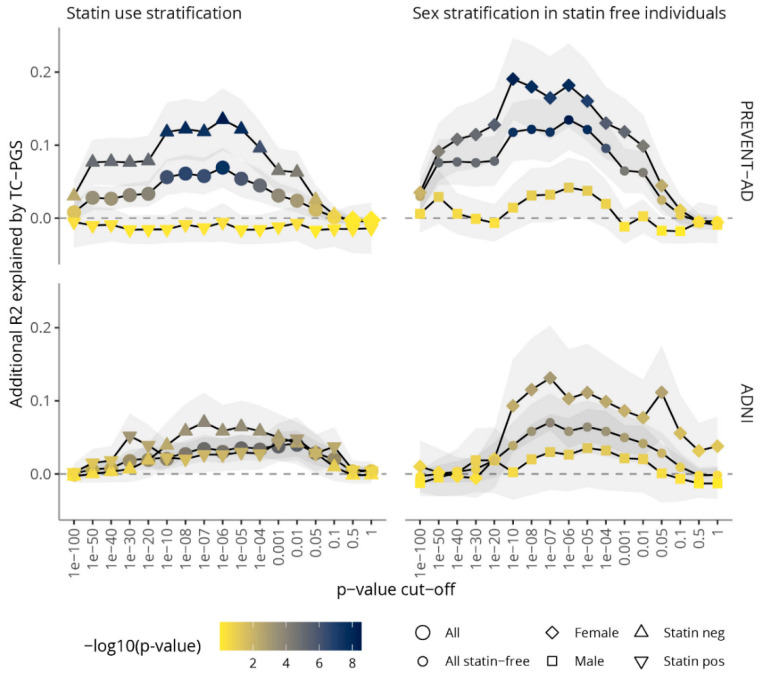
Variance explained by TC-PGS on TC levels over multiple *p*-value cut-offs. Multiple *p*-value thresholds were evaluated for association with TC levels in PREVENT-AD (upper panel) and ADNI (lower panel) stratified for statin use (left-hand panel) and sex (right-hand panel) using linear regression corrected for PCs 1–10, *APOE*-ε4 status, age, and age^2^. Statin use and sex were included when not stratified for. Plotted is additional variance explained (regression R^2^) after adding the TC-PGS to the model. Abbreviations: TC-PGS, total cholesterol polygenic score; TC, total cholesterol.

To establish a TC-PGS that best associates with blood TC levels, various *p*-value cut-offs were investigated in the PREVENT-AD and ADNI cohorts (Figure 1). The different scores were first evaluated in all individuals, correcting for covariates as well as statin use and sex (Figure 1, left-hand panel, circles). At best, the TC-PGS explained 6.9% of the variance in PREVENT-AD (*p* = 2.93 × 10^−8^, *p*-value cut-off 1 × 10^−6^) and 4.1% in ADNI (*p* = 7.1 × 10^−6^, *p*-value cut-off 0.01). Stratification on statin use (Figure 1, left-hand panel, triangles) revealed strong associations in statin free individuals in both cohorts, increasing the variance explained to 13.5% in PREVENT-AD (*p* = 2.83 × 10^−9^, *p*-value cut-off 1 × 10^−6^) and 7.1% in ADNI (*p* = 1.4 × 10^−4^, *p*-value cut-off 1 × 10^−7^). In contrast, the scores in general performed poorly in statin users with none of the scores significantly associated with TC levels in PREVENT-AD (p’s ≥ 0.412) and the best score in ADNI explaining 5.2% of the variance (*p* = 7.2 × 10^−4^, *p*-value cut-off 1 × 10^−30^).

Statin free individuals were further stratified on sex (Figure 1, right-hand panel, squares and diamonds), revealing a highly significant effect in females; at best, the TC-PGS explained 19.0% of the variance in PREVENT-AD (*p* = 6.70 × 10^−9^, *p*-value cut-off 1 × 10^−10^) and 13.1% of the variance in ADNI (*p* = 9.6 × 10^−4^, *p*-value cut-off 1 × 10^−7^).

In either cohort, no association between TC-PGS’s and TC levels could be found in statin-free males (*p*’s > 0.05). Based on its performance in the younger, combined PREVENT-AD cohort, the TC-PGS with a *p*-value cut-off of 1 × 10^−6^ were selected for further analyses and will from hereon be referred to solely as the “TC-PGS”.

### 3.3. TC-PGS Predicts Hypercholesterolemia

**Figure 2:**
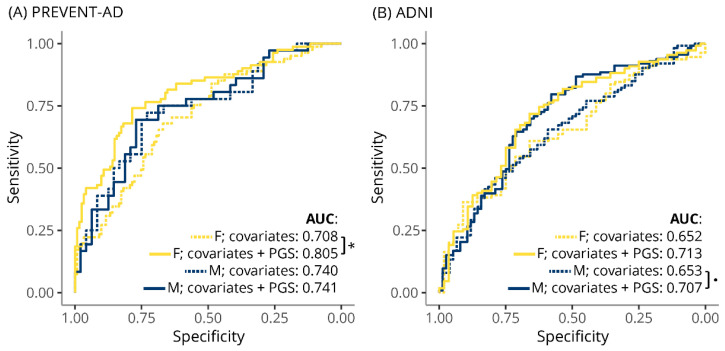
Effect of TC-PGS on prediction of hypercholesterolemia. ROC curves showing the effect of covariates and TC-PGS on predicting hypercholesterolemia in (**A**) PREVENT-AD and (**B**) ADNI, stratified for sex. Individuals were deemed hypercholesterolemic if they were on statins or TC levels > 6.2 mM (i.e., 240 mg/dL). Covariates included were genetic PCs 1–10, age, and age^2^. * *p* < 0.05, ∙ *p* < 0.1. Abbreviations: AUC, area under the curve; F, females; M, males; PC, genetic principal component; ROC, receiver operator characteristics; TC-PGS, total cholesterol polygenic score.

Next, we examined the TC-PGS’s ability to predict hypercholesterolemia in PREVENT-AD and ADNI (Figure 2). Receiver operator characteristics (ROC) curve analysis in PREVENT-AD revealed a significant improvement in hypercholesterolemia prediction in females with the addition of the TC-PGS to the model (area under the curve (AUC) increase from 70.8 to 80.5%, *p* = 0.0042) but no effect in males (AUC 74.0 vs. 74.1% in model without and with TC-PGS, respectively, *p* = 0.91). In ADNI, although adding the TC-PGS increased the AUC values for both females (65.2 vs. 71.3%, *p* = 0.14) and males (65.3 vs. 70.7%, *p* = 0.087), these increases did not reach significance.

### 3.4. TC-PGS Does Not Associate with Amyloid Pathology

**Figure 3:**
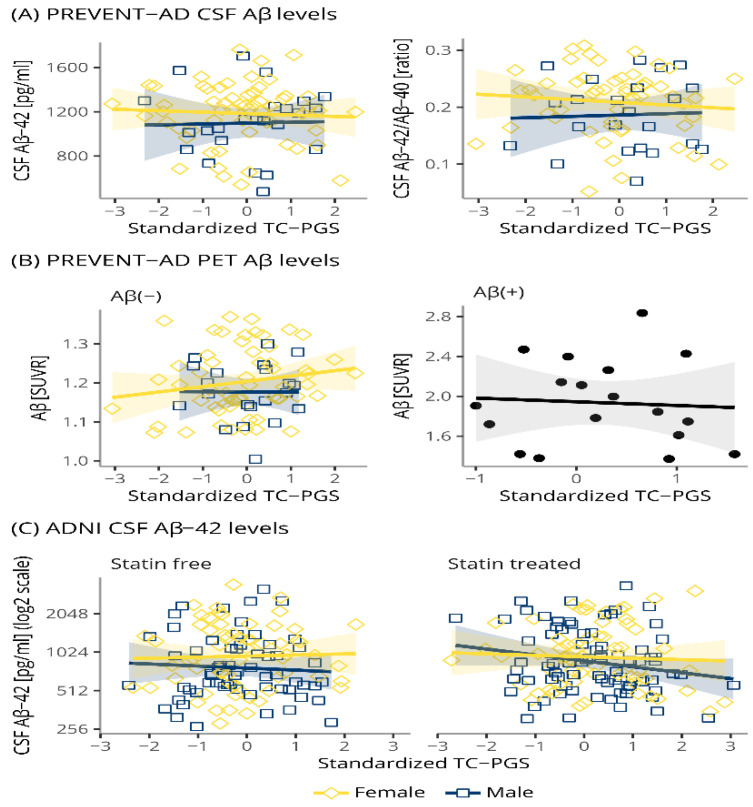
Associations of TC-PGS with biomarkers of amyloid pathology. Aβ pathology biomarkers were plotted against TC-PGS and assessed by multiple linear regressions. (**A**) PREVENT-AD CSF levels of Aβ-42 and its ratio with Aβ-40. (**B**) PREVENT-AD amyloid brain levels, as assessed by PET after stratification by Aβ(+) status (SUVR > 1.37). Due to the small sample size of Aβ(+) individuals (*n* = 18), only the main effect of TC-PGS was investigated. (**C**) ADNI CSF levels of Aβ-42. Shaded areas indicate 95% confidence intervals. Abbreviations: Aβ, amyloid-β; CSF, cerebrospinal fluid; PET, positron emission tomography; SUVR, standardized uptake value ratio; TC-PGS, total cholesterol polygenic score.

The effect of TC-PGS on Aβ pathology was assessed in PREVENT-AD and ADNI (Figure 3). In PREVENT-AD, linear regressions correcting for covariates and with a sex*TC-PGS interaction term revealed no effect of the TC-PGS, either as part of the interaction term or as a main effect, on CSF Aβ-42 (t_main_(16, 66) = −0.172, p_main_ = 0.864; t_int_(16, 66) = 0.269, p_int_ = 0.788), or its ratio with Aβ-40 (t_main_(16, 58) = −0.271, p_main_ = 0.787; t_int_(16, 58) = 0.024, p_int_ = 0.981). Similarly, in ADNI no significant effect of TC-PGS could be detected on CSF Aβ-42, neither as a main effect nor as part of any of the interaction terms (sex*TC-PGS, statin*TC-PGS, statin*sex*TC-PGS (−0.869 ≤ t’s(19, 250) ≤ 0.663, p’s ≥ 0.371). Further, the effect of TC-PGS was evaluated on Aβ pathology assessed by PET in PREVENT-AD. Due to not being normally distributed, the data was analyzed with a robust regression and by linear regression after stratifying for Aβ(+) status. We found no effect of the TC-PGS in the combined cohort or after stratification on Aβ(+) status (combined: t_main_(16, 80) = 0.898, p_main_ = 0.510, t_int_(16, 80) = −0.717, p_int_ = 0.703; Aβ(−): t_main_(16, 63) = 1.095, p_main_ = 0.278; t_int_(16, 63) = −0.672, p_int_ = 0.504; Aβ(+): t(5, 11) = −0.213, *p* = 0.8351).

### 3.5. TC-PGS Does Not Associate with TAU Pathology

**Figure 4:**
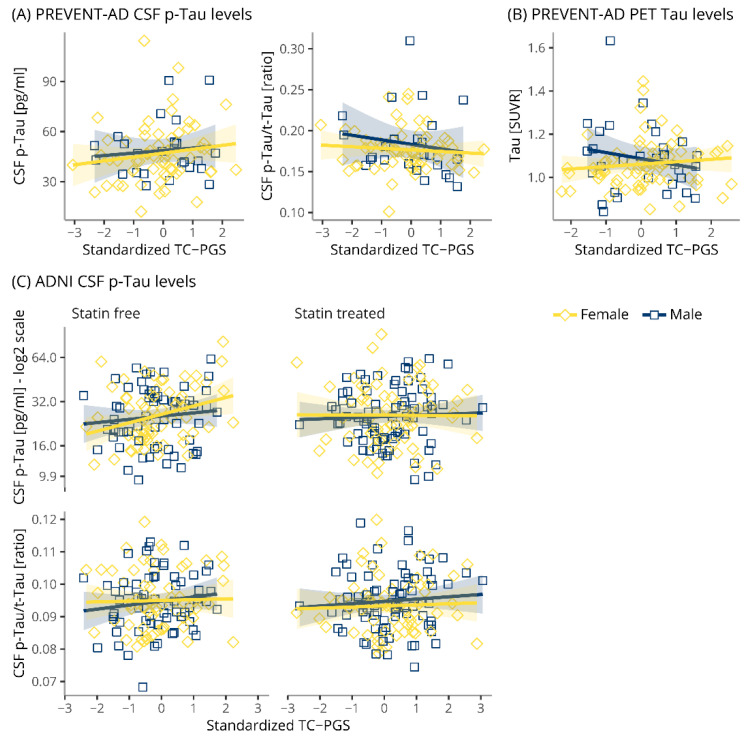
Associations of TC-PGS with biomarkers of TAU pathology. TAU pathology biomarkers plotted against TC-PGS and assessed by multiple linear regressions. (**A**) PREVENT-AD CSF levels of p-Tau and its ratio with t-Tau levels. (**B**) PREVENT-AD TAU brain levels, as assessed by PET. (**C**) ADNI CSF levels of *p*-Tau and its ratio with t-Tau. Shaded areas indicate 95% confidence intervals. Abbreviations: Aβ, amyloid-β; CSF, cerebrospinal fluid; PET, p-Tau, phosphorylated TAU; t-Tau, total TAU; positron emission tomography; SUVR, standardized uptake value ratio; TC-PGS, total cholesterol polygenic score.

The TC-PGS was evaluated for associations with biomarkers of TAU pathology in PREVENT-AD (CSF and PET) and ADNI (CSF, Figure 4). In PREVENT-AD, linear regressions corrected for covariates and with a sex*TC-PGS interaction revealed no associations between TC-PGS and biomarkers of TAU pathology as assessed by CSF *p*-Tau (t_main_(16, 69) = 0.641, p_main_ = 0.523; t_int_(16, 69) = −0.069, p_int_ = 0.946), CSF *p*-Tau/total TAU (t-Tau) ratio (t_main_(16, 69) = −0.919, p_main_ = 0.3614; t_int_(16, 69) = 0.819, p_int_ = 0.4155) and TAU PET (t_main_(16, 83) = −1.279, p_main_ = 0.204; t_int_(16, 83) = 1.641, p_int_ = 0.105). Similarly, in ADNI, we found no significant associations between the TC-PGS and TAU pathology as assessed by CSF p-Tau (−1.212 ≤ ts(19, 251) ≤ 1.247, p’s ≥ 0.214) and its ratio with t-Tau (−0.599 ≤ t’s(19, 251) ≤ 0.123, p’s ≥ 0.550).

### 3.6. TC-PGS Does Not Associate with Markers of Neurodegeneration

**Figure 5:**
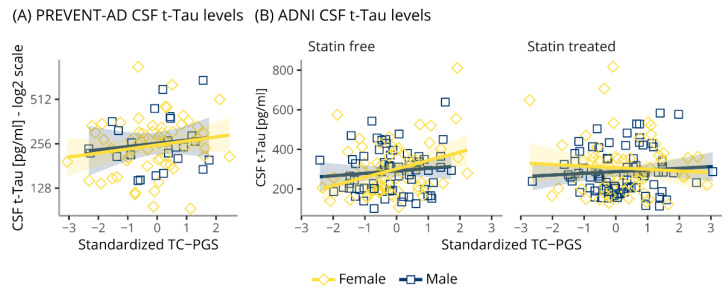
Associations of TC-PGS with biomarkers of neurodegeneration. Biomarkers of neurodegeneration as assessed by CSF levels of t-Tau plotted against TC-PGS. Data was analyzed by multiple linear regressions. (**A**) PREVENT-AD CSF levels of t-Tau. (**B**) ADNI CSF t-Tau levels. Shaded areas indicate 95% confidence intervals. Abbreviations: CSF, cerebrospinal fluid; t-Tau, total TAU; TC-PGS, total cholesterol polygenic score.

The TC-PGS was evaluated for associations with biomarkers of neurodegeneration in PREVENT-AD and ADNI by measuring levels of CSF t-Tau (Figure 5). We found no evidence for an association of the TC-PGS with CSF t-Tau in neither PREVENT-AD (t_main_(16, 69) = 0.758, p_main_ = 0.451; t_int_(16, 69) = −0.111, p_int_ = 0.912) nor ADNI (−1.393 ≤ t’s(19, 251) ≤ 1.533, p’s ≥ 0.127).

### 3.7. TC-PGS Does Not Associate with Increased Risk of Becoming Aβ(+)

The association between TC-PGS and risk of AD, defined as being Aβ(+), was evaluated in all three target cohorts (Table 2).

Individuals in ADNI and ROSMAP were categorized based on the presence of Aβ pathology in the brain as either Aβ(−) or Aβ(+) (see Method section for classification). We did not find any significant effect of TC-PGS on the risk of becoming Aβ(+) in neither PREVENT-AD, ADNI, nor ROSMAP. Stratification by statin used and sex did not lead to any significant association either.

### 3.8. TC-PGS Does Not Associate with Cognition in Aβ(+) Individuals

**Figure 6:**
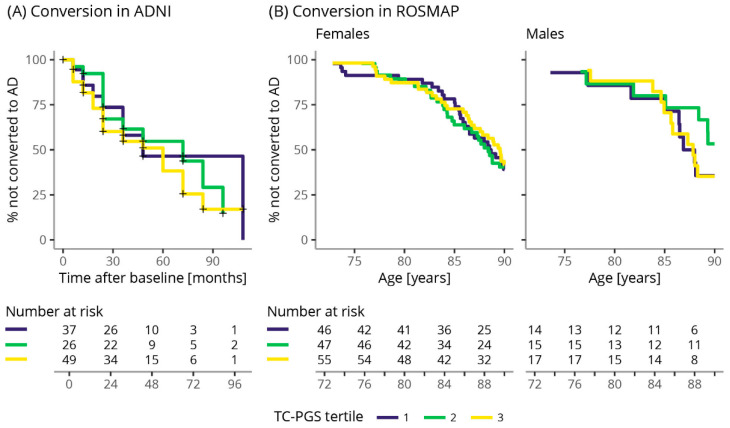
No effect of TC-PGS on AD conversion rate and age of onset. Kaplan–Meier survival curves displaying conversion rates to a clinical diagnosis of AD depending on TC-PGS tertiles in Aβ(+) individuals. (**A**) Conversion rate from healthy or MCI to AD as assessed by months after baseline visit in ADNI. (**B**) Conversion rate from healthy or MCI to AD as assessed by age in ROSMAP, stratified for sex. Abbreviations: AD, Alzheimer’s disease; TC-PGS, total cholesterol polygenic score.

Finally, we evaluated whether the TC-PGS is associated with the risk of becoming cognitively impaired in ADNI and ROSMAP (Table 3). For this analysis, we used the subset of individuals that were Aβ(+) and defined cognitive impairment (CI) as having any diagnosis of CI (e.g., including mild cognitive impairment (MCI), AD, and other dementias) at the last recorded visit. In neither ADNI nor ROSMAP could we detect any significant association between the TC-PGS and risk of becoming cognitively impaired. Stratification by sex does not lead to any significant associations. We also evaluated whether the TC-PGS had any effect on the conversion rate (ADNI, Figure 6A) or the age of onset (ROSMAP, Figure 6B). Aβ(+) individuals, either non-CI or with an MCI diagnosis at baseline, were selected as a subset of ADNI. The “event” was defined as receiving a clinical diagnosis of AD. Survival analysis revealed no difference between TC-PGS tertiles on conversion rate in ADNI (χ^2^(2) = 1.1, *p* = 0.6). In ROSMAP, we examined the association between TC-PGS tertiles and age at onset of a clinical diagnosis of possible or probable AD; however, we found no difference between the tertile groups (χ^2^(2) = 0.2, *p* = 0.9).

## 4. Discussion

### 4.1. TC-PGS and TC Levels

In this study, we have created a TC-PGS that associates with blood TC levels in two AD-related cohorts. We show that the variability explained by the score depends on the cohort, selection of SNPs to include in the score, statin use, and sex. We used clumping and *p*-value thresholding as a method for pruning SNPs to include in the scores, and thus, evaluated a number of *p*-value thresholds in both PREVENT-AD and in ADNI. We found that the score that explained most of the variance varied between the two cohorts and depended on statin use and sex stratification. For instance, a *p*-value threshold of 1 × 10^−6^ performed best in PREVENT-AD, while a threshold of 0.01 performed best in ADNI in the non-stratified analyses. In addition, the scores in general performed better in PREVENT-AD than in ADNI (Figure 1). Further, stratification on both statin use and sex had a remarkable effect on the scores’ performance in PREVENT-AD, and less so in ADNI. For example, in PREVENT-AD, the TC-PGSs had significant associations in statin-free females, with no significant associations in statin-treated individuals and males.

Similarly, examining the predictive ability of the TC-PGS on hypercholesterolemia revealed a significant improvement in PREVENT-AD females after the addition of the TC-PGS to the model, increasing AUC from 0.708 to 0.805 but not in males (Figure 2). In ADNI, we detected similar trends for improved AUCs in females and males, but this did not reach significance (p’s > 0.08).

The discrepancies between cohorts could possibly be due to the differences between PREVENT-AD and ADNI (Table 1) that differ in the proportion of females, *APOE*-ε4 carriers and statin users, as well as age. With sex and statin stratifications, we see that results do become more similar, further supporting the importance of taking these factors into account. Another factor that could affect the associations is that cholesterol measurements were taken after fasting in ADNI, whereas in PREVENT-AD, non-fasted samples were used, although studies have shown that TC levels are little influenced by fasting conditions [60,61].

The *APOE* gene locus is one of the most important for TC levels. The top SNP in the results from Willer et al. [24] is indeed rs7412—the SNP, together with rs429358, that determines the *APOE*-ε4 genotype. Its C allele combines to either result in the ε3 or ε4 alleles (as opposed to the ε2 allele) and associates with increased TC levels (β = 0.374, *p* = 1.560 × 10^−283^). Rs429358 further determines the ε4 allele and is not present in the summary data. Nevertheless, rs429358 has been shown to associate with TC levels in other big GWAS’s [62,63] such that the C allele, which results in the ε4 allele, associates with increased TC levels.

It should be noted that the two cohorts also differ in terms of age; ADNI being on average 10 years older than PREVENT-AD. TC levels increase from early life over midlife to late-life [64], however, it appears to be decreasing with age in older adults above 70 years [18,65]. This altered metabolism of cholesterol with age possibly involves different sets of genes and could thus explain why the TC-PGS behave differently in the two differently aged cohorts. This hypothesis, however, need to be further investigated using either longitudinal studies or cross-sectional studies covering a bigger range of ages. Considering that increased midlife levels of TC [3,4,5,8] are associated with increased risk of AD, it is interesting that our TC-PGS performs better in PREVENT-AD, which is closer to midlife than ADNI, thus suggesting it is better capturing midlife than late-life cholesterol levels.

The interaction between age and sex is of interest. For example, menopause in women is associated with increased TC levels and risk of cardiovascular disease [66,67] and hormone replacement therapy has been shown to decrease TC levels [68]. PREVENT-AD are younger and have a higher percentage of females compared to ADNI, and one could thus hypothesize those discrepancies in TC metabolism could also be influenced by discrepancies in the proportions of individuals that underwent menopause and treatment thereof.

Finally, compared to the study by Proitsi [28], our results show that the variance explained in blood TC levels by a TC-PGS can be vastly improved (3.6% in [28] vs. 18.2% for *p*-value cut-off 1 × 10^−6^ in statin free females in PREVENT-AD) by considering statin use and sex in the model.

### 4.2. TC-PGS and AD

Contrary to our significant findings between a TC-PGS and TC blood levels, the TC-PGS showed no associations with any biomarkers of AD pathologies (Figure 3 and Figure 4), neurodegeneration (Figure 5), or cognition (Figure 6, Table 3). Similarly, the TC-PGS did not associate with the risk of becoming Aβ(+) (Table 2), whether stratified by sex or statin use.

The relationship between vascular factors and AD biomarkers was recently assessed in PREVENT-AD and showed that vascular factors, including TC levels, associate with increased Aβ pathology, but only in individuals free of vascular medication, which include statins [13]. In contrast to the current study, where individuals were grouped based on statin use only, Köbe et al. included other medications relevant to cardiovascular disease (drugs against hypercholesterinemia and hypertension). Although samples size was an issue in PREVENT-AD, in ADNI, we had a sufficient sample size to include statin and sex use as interaction terms. Nevertheless, we could not find evidence for any association between the TC-PGS and AD biomarkers in both cohorts, maybe indicating that further vascular medications, rather than just statin use, need to be formally considered.

It is also possible that there is an additive effect of vascular risk factors such that the TC-PGS alone is not sufficient to have an effect on AD. Kivipelto et al. showed in multiple studies that there is an additive effect of TC levels, blood pressure, and *APOE*-ε4 [6,7,8], leading to the development of the cardiovascular risk factors, aging, and dementia (CAIDE) score [69]. This score takes into account age, sex, education, systolic blood pressure, body mass index, cholesterol, physical activity, and *APOE*-ε4 status and has been validated as a predictor for AD [70]. Similarly, vascular burden scores, taking into account factors such as hyperlipidemia, diabetes, and hypertension, are associated with impaired executive function and lower the threshold of amyloid burden needed to result in cognitive impairment [71]. This raises the important issue that concomitant vascular pathology may have severely confounded previous studies that established the link between mid-life total cholesterol and late-life AD risk. A note of interest, low education is associated with worse lipid profiles in women and better lipid profiles in men, the subgroup most susceptive to developing AD with aging. The percentages of intra-individual biological variability of total cholesterol, LDL and HDL do not exceed 9% in the normal population [72]. Complementary studies are now required to help to better understand the possible interplay between genetics and education pathways as they may both modulate AD risk in the elderly population where socioeconomic inequalities are quite common.

As mentioned above, *APOE* is important both for TC levels and AD risk. In this study design, we decided to keep the *APOE* gene locus in the TC-PGS but to correct for *APOE*-ε4 status in each regression model. Thus, the associations between the TC-PGS and TC levels are in addition to any effect of *APOE*-ε4 status. Similarly, the lack of association between TC-PGS and AD is after correcting for *APOE*-ε4 status. It is thus possible that the increased risk of AD seen in *APOE*-ε4 carriers is actually mediated in large part by independent processes found both in the periphery and in the CNS. For example, our group reported a surprisingly strong association between CSF concentrations of apolipoprotein B (apoB) and phospho(181)-tau in the pre-symptomatic phase of the disease in elderly subjects who are “at-risk” of AD because of a parental history. ApoB-containing lipoproteins such as LDL and VLDL have been associated with vascular or mixed dementia [73] in contrast to total cholesterol, which is the one clearly associated with AD risk. The observed apoB/phospho-tau association in pre-symptomatic AD is markedly modulated by the presence of the *APOE*-ε4 allele but not by the passage of peripheral apoB into the CNS [74]; supporting the notion that the total cholesterol could act more as a surrogate biomarker for *APOE*-ε4 mediated effects than a direct player in the pathophysiological process. This would be consistent with the above results showing that genetic variants, other than the genetic variants resulting in the *APOE*-ε4 isoform, strongly correlate with TC levels but fail to associate with AD pathology.

## 5. Conclusions

In summary, we have created a TC-PGS that associates with TC levels and significantly improves the prediction of hypercholesterolemia, specifically in statin-free females with European ancestry. We could, however, not prove any significant associations with AD, neither on the neuropathological underpinnings nor on cognition. It is possible that explaining ~18% of the variance in blood TC levels is still not enough to find significant associations with AD. For example, while it has previously been shown that TC levels are associated with Aβ pathology in PREVENT-AD [13], the TC-PGS was not in the same cohort, which would suggest that we would possibly need a bigger sample size. Furthermore, considering the fact that there is an additive effect of vascular risk factors on AD, it is still possible that the TC-PGS could have an effect on AD in individuals at higher cardiovascular risk (e.g., *APOE*-ε4 carriers). Further research is warranted to establish the role of a TC-PGS in AD.

## Figures and Tables

**Table 1:** Cohort characteristics.

	PREVENT-AD		ADNI		ROSMAP		
Variable	N	Mean (SE)	N	Mean (SE)	N	Mean (SE)	*p*-Value
Females [%]	306	69.9 (2.6)	401	47.9 (2.5)	547	71.1 (1.9)	<0.001 ^a^
APOE-ε4 carriers [%]	302	37.1 (2.8)	401	56.9 (2.5)	546	24.2 (1.8)	<0.001 ^a^
Age [years] ^#^	264	63.33 (0.42)	401	72.79 (0.35)	547	88.42 (0.13)	<0.001 ^b^
Statin treated [%]	299	23.4 (2.5)	360	51.4 (2.6)	*NA*	*NA*	<0.001 ^a^
TC measurements							
Age @ blood collection	287	63.15 (0.3)	355	72.63 (0.37)	*NA*	*NA*	<0.001 ^c^
TC [mM]	287	5.42 (0.06)	355	5.09 (0.06)	*NA*	*NA*	<0.001 ^c^
CSF measurements							
Age @ CSF collection	86	62.87 (0.59)	302	72.19 (0.41)	*NA*	*NA*	<0.001 ^c^
Aβ-42 [pg/ml]	83	1160.31 (31.08)	301	1053.78 (37.17)	*NA*	*NA*	0.029 ^c^
Aβ-40 [pg/ml]	75	6132.53 (222.57)	*NA*	*NA*	*NA*	*NA*	*NA*
p-Tau [pg/ml]	86	47.31 (1.89)	302	28.03 (0.76)	*NA*	*NA*	<0.001 ^c^
t-Tau [pg/ml]	86	275.23 (13.87)	302	291.45 (6.95)	*NA*	*NA*	0.298 ^c^
PET measurements							
Age @ Aβ PET [years]	98	67.62 (0.49)	*NA*	*NA*	*NA*	*NA*	*NA*
Aβ PET [SUVR]	98	1.33 (0.04)	*NA*	*NA*	*NA*	*NA*	*NA*
Age @ TAU PET [years]	100	70.79 (0.56)	*NA*	*NA*	*NA*	*NA*	*NA*
TAU PET [SUVR]	100	1.07 (0.01)	*NA*	*NA*	*NA*	*NA*	*NA*
Aβ(+)							
Age @ assessment [years]	98	67.62 (0.49)	271	74.22 (0.45)	535	88.38 (0.13)	<0.001 ^b^
Aβ(+) [%]	98	18.4 (0.04)	271	54.2 (3.0)	535	74.6 (1.9)	<0.001 ^a^
Cognition							
Age @ assessment [years]	*NA*	*NA*	139	76.62 (0.63)	399	88.5 (0.15)	<0.001 ^c^
CI [%]	*NA*	*NA*	139	90.6 (2.5)	399	76.9 (2.1)	<0.001 ^a^

Abbreviations: Aβ, amyloid-β; CI, cognitively impaired; CSF, cerebrospinal fluid; p-Tau, phosphorylated TAU; PET, positron emission tomography; SE, standard error of the mean; SUVR, standardized uptake value ratio; TC, total cholesterol. ^#^ Mean age was calculated for baseline in PREVENT-AD and ADNI, and for age of death in ROSMAP. ^a^ Calculated with χ^2^ test. ^b^ Calculated with ANOVA. ^c^ Calculated with Welch two sample *t*-test.

**Table 2:** TC-PGS effect on amyloid positivity status.

	PREVENT-AD		ADNI		ROSMAP	
TC-PGS terms	ORs	*p*-Value	ORs	*p*-Value	ORs	*p*-Value
TC-PGS	0.50 (0.07–3.24)	0.25	0.82 (0.40–1.66)	0.58	0.95 (0.66–1.37)	0.77
Statin use*TC-PGS	NA	NA	1.68 (0.31–1.91)	0.27	NA	NA
Sex*TC-PGS	3.64 (0.42–39.4)	0.25	1.08 (0.20–1.25)	0.87	0.87 (0.56-1.35)	0.54
Statin use*Sex* TC-PGS	NA	NA	0.60 (0.05–0.53)	0.41	NA	NA

ORs for interaction terms refer to statin treated, females with an increase in 1 SD of TC-PGS. NA indicates that interaction was not investigated due to insufficient sample size. 95% confidence intervals shown within brackets (). Abbreviations: Aβ(+), amyloid-β positivity as assessed by CSF p-Tau/Aβ-42 ratio (≥ 0.028); CI, cognitively impaired (only Aβ(+) individuals); OR, odds ratio; TC-PGS, total cholesterol polygenic score.

**Table 3:** TC-PGS effect on cognitive impairment.

	ADNI		ROSMAP	
TC-PGS Terms	ORs	*p*-Value	ORs	*p*-Value
TC-PGS	1.08 (0.44–2.69)	0.86	0.76 (0.43–1.33)	0.34
Sex*TC-PGS	1.18 (0.38–3.89)	0.78	1.40 (0.76–2.61)	0.28

ORs for interaction terms refer to statin treated, females with an increase in 1 SD of TC-PGS. 95% confidence intervals shown within brackets (). Abbreviations: Aβ(+), amyloid-β positivity as assessed by CSF p-Tau/Aβ-42 ratio (≥0.028); CI, cognitively impaired (only Aβ(+) individuals); OR, odds ratio; TC-PGS, total cholesterol polygenic score.

## Data Availability

PREVENT-AD data is available upon request to the authors via the research centre’s website: StoP-AD Centre (prevent-alzheimer.net, accessed on 26 January 2018). For all other datasets, please refer to the Material and Methods Section. Data used in the preparation of this article were obtained from the ADNI database (adni.loni.usc.edu, accessed on 3 December 2015). As such, the investigators within the ADNI contributed to the design and implementation of ADNI and/or provided data but did not participate in the analysis or writing of this report. A complete listing of ADNI investigators can be found at: adni.loni.usc.edu/wp-content/uploads/how_to_apply/ADNI_Acknowledgement_List.pdf (accessed on 22 June 2018).

## Supplementary Materials

The following are available online at https://www.mdpi.com/article/10.3390/genes12111805/s1, Table S1: Datasets, software, and R packages. Figure S1: Manhattan plots of included/excluded SNPs.

## References

[1] Alzheimer’s Association (2017). 2017 Alzheimer’s disease facts and figures. Alzheimer’s Dement. J. Alzheimer’s Assoc..

[2] Ridge P.G., Hoyt K.B., Boehme K., Mukherjee S., Crane P.K., Haines J.L., Mayeux R., Farrer L., Pericak-Vance M.A., Schellenberg G.D. (2016). Assessment of the genetic variance of late-onset Alzheimer’s disease. Neurobiol. Aging.

[3] Reiman E.M., Chen K., Langbaum J.B., Lee W., Reschke C., Bandy D., Alexander G.E., Caselli R.J. (2010). Higher serum total cholesterol levels in late middle age are associated with glucose hypometabolism in brain regions affected by Alzheimer’s disease and normal aging. NeuroImage.

[4] Solomon A., Kivipelto M., Wolozin B., Zhou J., Whitmer R.A. (2009). Midlife Serum Cholesterol and Increased Risk of Alzheimer’s and Vascular Dementia Three Decades Later. Dement. Geriatr. Cogn. Disord..

[5] Toro P., Degen C., Pierer M., Gustafson D., Schröder J., Schönknecht P. (2014). Cholesterol in mild cognitive impairment and Alzheimer’s disease in a birth cohort over 14 years. Eur. Arch. Psychiatry Clin. Neurosci..

[6] Kivipelto M., Helkala E.-L., Laakso M., Hänninen T., Hallikainen M., Alhainen K., Soininen H., Tuomilehto J., Nissinen A. (2001). Midlife vascular risk factors and Alzheimer’s disease in later life: Longitudinal, population based study. BMJ.

[7] Kivipelto M., Helkala E.-L., Laakso M., Hänninen T., Hallikainen M., Alhainen K., Iivonen S., Mannermaa A., Tuomilehto J., Nissinen A. (2002). Apolipoprotein E ϵ4 Allele, Elevated Midlife Total Cholesterol Level, and High Midlife Systolic Blood Pressure Are Independent Risk Factors for Late-Life Alzheimer Disease. Ann. Intern. Med..

[8] Kivipelto M., Ngandu T., Fratiglioni L., Viitanen M., Kåreholt I., Winblad B., Helkala E.-L., Tuomilehto J., Soininen H., Nissinen A. (2005). Obesity and Vascular Risk Factors at Midlife and the Risk of Dementia and Alzheimer Disease. Arch. Neurol..

[9] Li G., Shofer J.B., Kukull W.A., Peskind E.R., Tsuang D.W., Breitner J., McCormick W., Bowen J.D., Teri L., Schellenberg G.D. (2005). Serum cholesterol and risk of Alzheimer disease: A community-based cohort study. Neurology.

[10] Tan Z.S., Seshadri S., Beiser A., Wilson P.W.F., Kiel D., Tocco M., D’Agostino R.B., Wolf P.A. (2003). Plasma Total Cholesterol Level as a Risk Factor for Alzheimer Disease. Arch. Intern. Med..

[11] Mielke M.M., Zandi P.P., Shao H., Waern M.M., Östling S., Guo X.M., Björkelund C., Lissner L., Skoog I.M. (2010). The 32-year relationship between cholesterol and dementia from midlife to late life. Neurology.

[12] Pappolla M.A., Bryant-Thomas T., Herbert D., Pacheco J., Garcia M.F., Manjon M., Gironès X., Henry T., Matsubara E., Zambon D. (2003). Mild hypercholesterolemia is an early risk factor for the development of Alzheimer amyloid pathology. Neurology.

[13] Köbe T., Gonneaud J., Binette A.P., Meyer P.-F., McSweeney M., Rosa-Neto P., Breitner J.C.S., Poirier J., Villeneuve S. (2020). For the Presymptomatic Evaluation of Experimental or Novel Treatments for Alzheimer Disease (PREVENT-AD) Research Group Association of Vascular Risk Factors With β-Amyloid Peptide and Tau Burdens in Cognitively Unimpaired Individuals and Its Interaction With Vascular Medication Use. JAMA Netw. Open.

[14] Reed B., Villeneuve S., Mack W., DeCarli C., Chui H.C., Jagust W. (2014). Associations Between Serum Cholesterol Levels and Cerebral Amyloidosis. JAMA Neurol..

[15] Lesser G., Kandiah K., Libow L., Likourezos A., Breuer B., Marin D., Mohs R., Haroutunian V., Neufeld R. (2001). Elevated Serum Total and LDL Cholesterol in Very Old Patients with Alzheimer’s Disease. Dement. Geriatr. Cogn. Disord..

[16] Lesser G.T., Haroutunian V., Purohit D.P., Beeri M.S., Schmeidler J., Honkanen L., Neufeld R., Libow L.S. (2009). Serum Lipids Are Related to Alzheimer’s Pathology in Nursing Home Residents. Dement. Geriatr. Cogn. Disord..

[17] Sabbagh M., Zahiri H.R., Ceimo J., Cooper K., Gaul W., Connor D., Sparks D.L. (2005). Is there a characteristic lipid profile in Alzheimer’s disease?. J. Alzheimer’s Dis..

[18] Mielke M.M., Zandi P.P., Sjogren M., Gustafson D., Ostling S., Steen B., Skoog I. (2005). High total cholesterol levels in late life associated with a reduced risk of dementia. Neurology.

[19] Rabinovici G.D., Carrillo M.C., Forman M., DeSanti S., Miller D.S., Kozauer N., Petersen R.C., Randolph C., Knopman D.S., Smith E.E. (2017). Multiple comorbid neuropathologies in the setting of Alzheimer’s disease neuropathology and implications for drug development. Alzheimer’s Dementia: Transl. Res. Clin. Interv..

[20] McKhann G.M., Knopman D.S., Chertkow H., Hyman B.T., Jack C.R., Kawas C.H., Klunk W.E., Koroshetz W.J., Manly J.J., Mayeux R. (2011). The diagnosis of dementia due to Alzheimer’s disease: Recommendations from the National Institute on Aging-Alzheimer’s association workgroups on diagnostic guidelines for Alzheimer’s disease. Alzheimers Dement..

[21] Jack C.R., Bennett D.A., Blennow K., Carrillo M.C., Dunn B., Haeberlein S.B., Holtzman D.M., Jagust W., Jessen F., Karlawish J. (2018). NIA-AA Research Framework: Toward a biological definition of Alzheimer’s disease. Alzheimer’s Dement..

[22] Clifford R.J., Bennett D.A., Blennow K., Carrillo M.C., Feldman H.H., Frisoni G.B., Hampel H., Jagust W.J., Johnson K.A., Knopman D.S. (2016). A/T/N: An unbiased descriptive classification scheme for Alzheimer disease biomarkers. Neurology.

[23] (2009). The ENGAGE Consortium Loci influencing lipid levels and coronary heart disease risk in 16 European population cohorts. Nat. Genet..

[24] Willer C.J., Schmidt E.M., Sengupta S., Peloso G.M., Gustafsson S., Kanoni S., Ganna A., Chen J., Buchkovich M.L., Mora S. (2013). Discovery and refinement of loci associated with lipid levels. Nat. Genet..

[25] Teslovich T.M., Musunuru K., Smith A.V., Edmondson A.C., Stylianou I.M., Koseki M., Pirruccello J.P., Ripatti S., Chasman D.I., Willer C.J. (2010). Biological, clinical and population relevance of 95 loci for blood lipids. Nature.

[26] Gatz M., Reynolds C.A., Fratiglioni L., Johansson B., Mortimer J.A., Berg S., Fiske A., Pedersen N.L. (2006). Role of Genes and Environments for Explaining Alzheimer Disease. Arch. Gen. Psychiatry.

[27] Heller D.A., De Faire U., Pedersen N.L., Dahlén G., McClearn G.E. (1993). Genetic and Environmental Influences on Serum Lipid Levels in Twins. N. Engl. J. Med..

[28] Proitsi P., Lupton M., Velayudhan L., Newhouse S., Fogh I., Tsolaki M., Daniilidou M., Pritchard M.R., Kloszewska I., Soininen H. (2014). Genetic Predisposition to Increased Blood Cholesterol and Triglyceride Lipid Levels and Risk of Alzheimer Disease: A Mendelian Randomization Analysis. PLoS Med..

[29] Breitner J.C.S., Poirier J., Etienne P.E., Leoutsakos J.M. (2016). Rationale and Structure for a New Center for Studies on Prevention of Alzheimer’s Disease (StoP-AD). J. Prev. Alzheimers Dis..

[30] Bennett D.A., Buchman A.S., Boyle P.A., Barnes L.L., Wilson R.S., Schneider J.A. (2018). Religious Orders Study and Rush Memory and Aging Project. J. Alzheimer’s Dis..

[31] Weale M.E. (2010). Quality Control for Genome-Wide Association Studies. MAP Kinase Signaling Protocols.

[32] Chang C.C., Chow C.C., Tellier L.C.A.M., Vattikuti S., Purcell S.M., Lee J.J. (2015). Second-generation PLINK: Rising to the challenge of larger and richer datasets. GigaScience.

[33] Purcell S., Neale B., Todd-Brown K., Thomas L., Ferreira M.A.R., Bender D., Maller J., Sklar P., de Bakker P.I.W.D., Daly M.J. (2007). PLINK: A Tool Set for Whole-Genome Association and Population-Based Linkage Analyses. Am. J. Hum. Genet..

[34] Sudmant P.H., Rausch T., Gardner E.J., Handsaker E.R., Abyzov A., Huddleston J., Zhang Y., Ye K., Jun G., Fritz M.H.-Y. (2015). An integrated map of structural variation in 2504 human genomes. Nature.

[35] Auton A., Brooks L.D., Durbin R.M., Garrison E.P., Kang H.M., Korbel J.O., Marchini J.L., McCarthy S., McVean G.A., The 1000 Genomes Project Consortium (2015). A global reference for human genetic variation. Nature.

[36] McCarthy S., Das S., Kretzschmar W., Delaneau O., Wood A.R., Teumer A., Kang H.M., Fuchsberger C., Danecek P., Sharp K. (2016). A reference panel of 64,976 haplotypes for genotype imputation. Nat. Genet..

[37] Delaneau O., Marchini J., Zagury J.-F. (2011). A linear complexity phasing method for thousands of genomes. Nat. Methods.

[38] Durbin R. (2014). Efficient haplotype matching and storage using the positional Burrows-Wheeler transform (PBWT). Bioinformatics.

[39] Verma S.S., de Andrade M., Tromp G., Kuivaniemi H., Epugh E., Namjou-Khales B., Mukherjee S., Jarvik G.P., Kottyan L., Eburt A. (2014). Imputation and quality control steps for combining multiple genome-wide datasets. Front. Genet..

[40] Meyer P.-F., Tremblay-Mercier J., Leoutsakos J., Madjar C., Lafaille-Maignan M.-É., Savard M., Rosa-Neto P., Poirier J., Etienne P., Breitner J. (2019). INTREPAD. Neurology.

[41] Bittner T., Zetterberg H., Teunissen C.E., Ostlund R.E., Militello M., Andreasson U., Hubeek I., Gibson D., Chu D.C., Eichenlaub U. (2016). Technical performance of a novel, fully automated electrochemiluminescence immunoassay for the quantitation of β-amyloid (1–42) in human cerebrospinal fluid. Alzheimer’s Dement..

[42] Shaw L.M., Hansson O., Manuilova E., Masters C.L., Doecke J.D., Li Q.-X., Rutz S., Widmann M., Leinenbach A., Blennow K. (2019). Method comparison study of the Elecsys® β-Amyloid (1–42) CSF assay versus comparator assays and LC-MS/MS. Clin. Biochem..

[43] McSweeney M., Binette A.P., Meyer P.-F., Gonneaud J., Bedetti C., Ozlen H., Labonté A., Rosa-Neto P., Breitner J., Poirier J. (2020). Intermediate flortaucipir uptake is associated with Aβ-PET and CSF tau in asymptomatic adults. Neurology.

[44] Hansson O., Seibyl J., Stomrud E., Zetterberg H., Trojanowski J.Q., Bittner T., Lifke V., Corradini V., Eichenlaub U., Batrla R. (2018). CSF biomarkers of Alzheimer’s disease concord with amyloid-β PET and predict clinical progression: A study of fully automated immunoassays in BioFINDER and ADNI cohorts. Alzheimer’s Dement..

[45] P Core Team (2021). R: A Language and Environment for Statistical Computing.

[46] Dowle M., Srinivasan A., Gorecki J., Chirico M., Stetsenko P., Short T., Lianoglou S., Antonyan E., Bonsch M., Parsonage H. Data.Table: Extension of ‘Data.Frame’. https://CRAN.R-project.org/package=data.table.

[47] Wickham H., Averick M., Bryan J., Chang W., McGowan L.D., François R., Grolemund G., Hayes A., Henry L., Hester J. (2019). Welcome to the Tidyverse. J. Open Source Softw..

[48] Wilke C.O. (2019). Cowplot: Streamlined Plot Theme and Plot Annotations for “ggplot2”. https://CRAN.R-project.org/package=cowplot.

[49] Revelle W. (2017). Psych: Procedures for Personality and Psychological Research.

[50] Canty A., Ripley B.D. (2020). Boot: Bootstrap R (S-Plus) Functions.

[51] Davison A.C., Hinkley D.V. (1997). Bootstrap Methods and Their Applications.

[52] Mangiafico S. (2020). Rcompanion: Functions to Support Extension Education Program Evaluation. https://CRAN.R-project.org/package=rcompanion.

[53] Robin X., Turck N., Hainard A., Tiberti N., Lisacek F., Sanchez J.-C., Muller M.J. (2011). pROC: An open-source package for R and S+ to analyze and compare ROC curves. BMC Bioinform..

[54] Rich J.T., Neely J.G., Paniello R.C., Voelker C.C.J., Nussenbaum B., Wang E. (2010). A practical guide to understanding Kaplan-Meier curves. Otolaryngol. Neck Surg..

[55] Therneau T.M., Grambsch P.M. (2000). Modeling Survival Data: Extending the Cox Model.

[56] Therneau T.M. (2020). A Package for Survival Analysis in R. https://CRAN.R-project.org/package=survival.

[57] Horikoshi M., Tang Y. (2016). Ggfortify: Data Visualization Tools for Statistical Analysis Results. https://CRAN.R-project.org/package=ggfortify.

[58] Tang Y., Horikoshi M., Li W. (2016). ggfortify: Unified Interface to Visualize Statistical Results of Popular R Packages. R J..

[59] Kassambara A., Kosinski M. (2016). Survminer: Drawing Survival Curves Using “ggplot2”. https://CRAN.R-project.org/package=survminer.

[60] Langsted A., Nordestgaard B.G. (2019). Nonfasting versus fasting lipid profile for cardiovascular risk prediction. Pathology.

[61] Grundy S.M., Stone N.J., Bailey A.L., Beam C., Birtcher K.K., Blumenthal R.S., Braun L.T., De Ferranti S., Faiella-Tommasino J., Forman D.E. (2019). 2018 AHA/ACC/AACVPR/AAPA/ABC/ACPM/ADA/AGS/APhA/ASPC/NLA/PCNA Guideline on the Management of Blood Cholesterol: A Report of the American College of Cardiology/American Heart Association Task Force on Clinical Practice Guidelines. J. Am. Coll. Cardiology..

[62] Hoffmann T.J., Theusch E., Haldar T., Ranatunga D.K., Jorgenson E., Medina M.W., Kvale M.N., Kwok P.-Y., Schaefer C., Krauss R.M. (2018). A large electronic-health-record-based genome-wide study of serum lipids. Nat. Genet..

[63] Kanai M., Akiyama M., Takahashi A., Matoba N., Momozawa Y., Ikeda M., Iwata N., Ikegawa S., Hirata M., Matsuda K. (2018). Genetic analysis of quantitative traits in the Japanese population links cell types to complex human diseases. Nat. Genet..

[64] Morgan A.E., Mooney K.M., Wilkinson S.J., Pickles N.A., Mc Auley M.T. (2017). Investigating cholesterol metabolism and ageing using a systems biology approach. Proc. Nutr. Soc..

[65] Jacobs J.M., Cohen A., Ein-Mor E., Stessman J. (2013). Cholesterol, Statins, and Longevity From Age 70 to 90 Years. J. Am. Med. Dir. Assoc..

[66] Ambikairajah A., Walsh E., Cherbuin N. (2019). Lipid profile differences during menopause: A review with meta-analysis. Menopause.

[67] Inaraja V., Thuissard I., Andreu-Vazquez C., Jodar E. (2020). Lipid profile changes during the menopausal transition. Menopause.

[68] Notelovitz M. (1976). The effect of long-term oestrogen replacement therapy on glucose and lipid metabolism in postmenopausal women. South Afr. Med. J..

[69] Kivipelto M., Ngandu T., Laatikainen T., Winblad B., Soininen H., Tuomilehto J. (2006). Risk score for the prediction of dementia risk in 20 years among middle aged people: A longitudinal, population-based study. Lancet Neurol..

[70] Exalto L.G., Quesenberry C.P., Barnes D., Kivipelto M., Biessels G.J., Whitmer R.A. (2014). Midlife risk score for the prediction of dementia four decades later. Alzheimer’s Dement..

[71] DeCarli C., Villeneuve S., Maillard P., Harvey D., Singh B., Carmichael O., Fletcher E., Olichney J., Farias S., Jagust W. (2019). Vascular Burden Score Impacts Cognition Independent of Amyloid PET and MRI Measures of Alzheimer’s Disease and Vascular Brain Injury. J. Alzheimer’s Dis..

[72] Lara M., Amigo H. (2018). Association between education and blood lipid levels as income increases over a decade: A cohort study. BMC Public Health.

[73] Schilling S., Tzourio C., Soumaré A., Kaffashian S., Dartigues J.-F., Ancelin M.-L., Samieri C., Dufouil C., Debette S. (2017). Differential associations of plasma lipids with incident dementia and dementia subtypes in the 3C Study: A longitudinal, population-based prospective cohort study. PLoS Med..

[74] Picard C., Nilsson N., Labonté A., Auld D., Rosa-Neto P., Ashton N.J., Zetterberg H., Blennow K., Breitner J.C., Villeneuve S. (2021). Apolipoprotein B is a novel marker for early tau pathology in Alzheimer’s disease. Alzheimer’s Dement..

