# Supplementary material for: Association of a Total Cholesterol Polygenic Score with Cholesterol Levels and Pathological Biomarkers across the Alzheimer’s Disease Spectrum
Source: Genes (Basel). 2021 Nov 17;12(11):1805. doi: 10.3390/genes12111805 (PMC8623969; doi:10.3390/genes12111805)
Supplement: Supplementary file 1 [file genes-12-01805-s001.zip › genes-1440986-supplementary.pdf]

Supporting information

Supplementary Table S1. Data sets, software and R packages.

Data set/software/R package	Link & references
**Data**	
1000 genomes	https://www.internationalgenome.org/ [47]
ADNI	http://adni.loni.usc.edu/
GLGC	http://csg.sph.umich.edu/willer/public/lipids2013/ [24]
GRCh37 genome	https://www.ncbi.nlm.nih.gov/assembly/GCF_000001405.13/#/st
PREVENT-AD	https://openpreventad.loris.ca/ [29]
ROSMAP	https://www.radc.rush.edu/ [30]
StoP-AD centre	https://douglas.research.mcgill.ca/stop-ad-centre [29]
**Software**	
PLINK	https://www.cog-genomics.org/plink2 [44,45]
R	https://www.r-project.org/ [57]
Sanger Imputation Service	https://imputation.sanger.ac.uk/ [48]
**R packages**	
boot	[62,63]
cowplot	https://CRAN.R-project.org/package=cowplot/ [60]
data.table	https://CRAN.R-project.org/package=data.table [58]
ggfortify	https://CRAN.R-project.org/package=ggfortify/ [69,70]
pROC	[65]
psych	https://CRAN.R-project.org/package=psych/ [61]
rcompanion	https://CRAN.R-project.org/package=rcompanion/ [64]
survival	[67,68]
survminer	https://CRAN.R-project.org/package=survminer/ [71]
tidyverse	https://www.tidyverse.org/ [59]

Supplementary Figure S1. Manhattan plots of included/excluded SNPs.

(A) Included SNPs

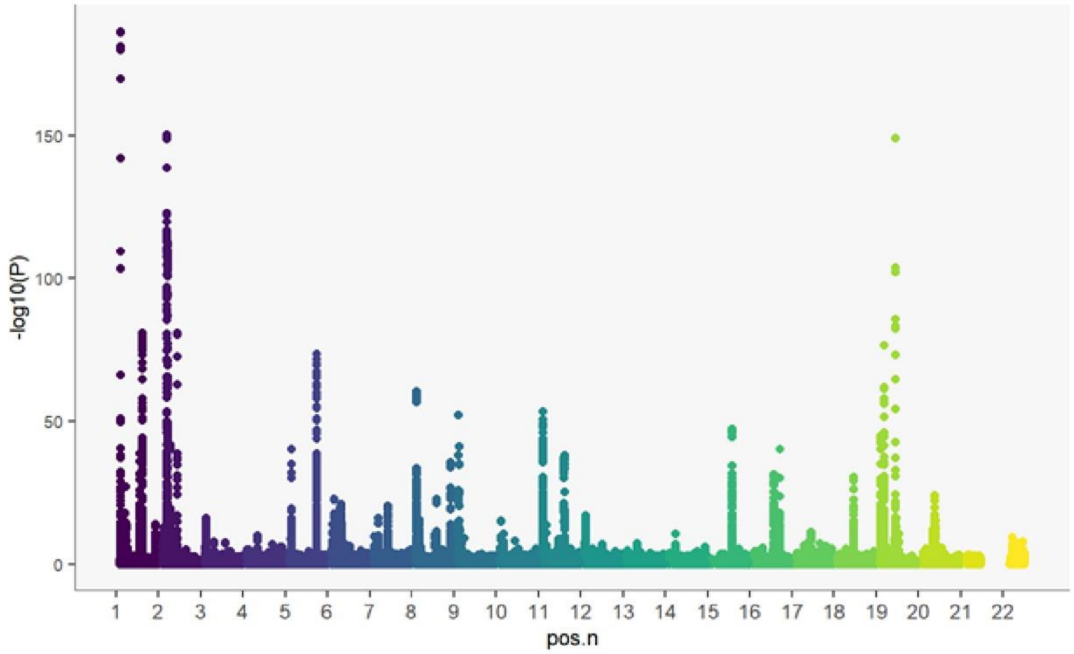

(B) Excluded SNPs

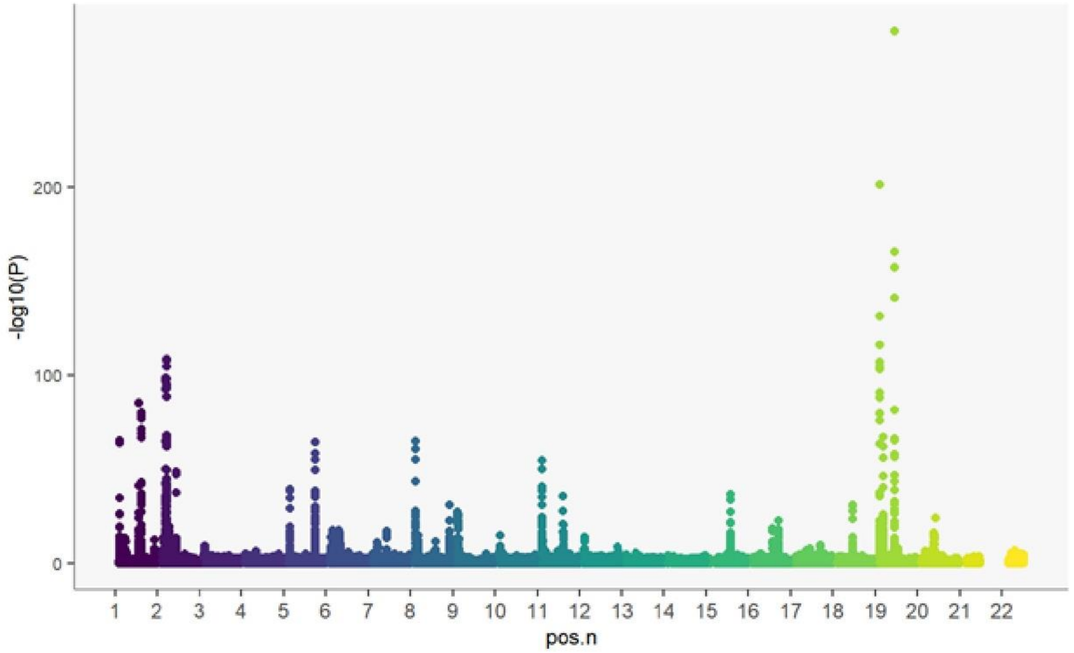

Supplementary Figure S1. Manhattan plots of included/excluded SNPs. Manhattan plots of SNPs evaluated for the TC-PGS (A) and excluded (B) after matching with the three target cohorts.
